# Health facility and contextual correlates of HIV test positivity: a multilevel model of routine programmatic data from Malawi

**DOI:** 10.1136/bmjph-2025-002568

**Published:** 2025-09-08

**Authors:** Miyu Niwa, Dylan Green, Tyler Smith, Brandon Klyn, Yohane Kamgwira, Sara Allinder, Deborah Hoege, Suzike Likumbo, Charles B Holmes, Gift Kawalazira, Linley Chewere

**Affiliations:** 1 Cooper/Smith, Austin, Texas, USA; 2 National AIDS Commission, Lilongwe, Malawi; 3 Center for Innovation in Global Health, Georgetown University, Washington, District of Columbia, USA; 4 Blantyre District Health Office, Blantyre District Council, Lilongwe, Malawi; 5 Department of HIV, STI & Hepatitis, Ministry of Health, Lilongwe, Malawi

**Keywords:** Sexually Transmitted Diseases, HIV, Epidemiology, Public Health

## Abstract

**Background:**

Innovative and efficient methods are needed to identify remaining people living with HIV unaware of their status. Routine health information system (RHIS) data, widely available in high-burden HIV settings, may help target areas of high risk to deliver timely prevention services. Often underused, RHIS data were leveraged at the facility level to predict changes in HIV test positivity in Malawi.

**Methods:**

From District Health Information Software-2 from January 2017 to March 2023, we analysed sexually transmitted infection (STI) cases and HIV tests and test results across 563 health facilities in Malawi. A multilevel model was employed to determine whether changes in STI diagnoses were predictive of changes in HIV test positivity. We considered STI types and their incubation periods, and controlled for facility type, ownership, quarter, season, zonal HIV and STI prevalence (2016 Population-Based HIV Impact Assessment).

**Results:**

Among 139 million HIV tests, overall positivity was 2.8%. Blantyre facilities had the highest positivity (6.0%) while those in the central-east zone had the lowest (1.8%). Key variables—changes in syndromic STI counts (lagged and cross-sectional)—showed weak or no associations with HIV positivity (OR: 1.01, CI: 1.01 to 1.01; OR: 1.00, CI: 1.00 to 1.00). However, contextual covariates, including zonal HIV prevalence (OR: 1.04, CI: 1.04 to 1.04), genital ulcers (OR: 1.16, CI: 1.16 to 1.16) and clinical STI diagnoses (OR: 1.29, CI: 1.29 to 1.29), were positively associated with HIV positivity.

**Conclusions:**

In settings with high STI screening uptake, RHIS data can be used to monitor changes in STI diagnoses and contextual factors to identify HIV hotspots and guide targeted testing, prevention and treatment services.

**WHAT IS ALREADY KNOWN ON THIS TOPIC**

- Studies identifying undiagnosed people living with HIV are often cross-sectional, using population-based surveys rather than routinely collected national programmatic data. An opportunity exists to use routine programme data for insights to inform service delivery and reach the ‘95-95-95’ HIV epidemic targets.

**WHAT THIS STUDY ADDS**

- Evidence for the association between changes in sexually transmitted infection diagnoses and changes in programmatic HIV test positivity at the facility level.

**HOW THIS STUDY MIGHT AFFECT RESEARCH, PRACTICE OR POLICY**

- Programmatic, routinely collected national data may be leveraged for HIV prevention-related resource allocation and prioritisation practice, as well as HIV hotspot identification, in the future.

## Introduction

 Malawi has made significant progress towards the 2025 United Nations Joint Programme on HIV/AIDS ‘95-95-95’ goals, with 88% of people living with HIV (PLHIV) being aware of their status.^1^ To reach the first target, an additional 56 385 PLHIV (estimated) need to be made aware of their HIV status. Social outreach programmes exist to identify more PLHIV, though many are currently driven by individual risk factors and thus target key populations.^1 2^ Standard HIV clinical testing and partner referral in high-endemicity environments are failing to yield the number of positive persons tested in the general population.^2^ Therefore, innovative, accurate and efficient methods are needed as the remaining PLHIV with unknown status becomes harder to identify.

A strategy to identify those living with HIV includes using routine health information system (RHIS) data for geospatial targeting of areas of high risk of infection at the right time to deliver prevention services and avert cases. In low-income and low-middle-income countries, RHISs have been implemented to support resource allocation and day-to-day decisions at multiple health system levels, and substantial investments have been made to develop, strengthen and digitise them.^3 4^ While researchers have preferred the use of cross-sectional, population-based surveys rather than RHIS data to conduct studies, there has been increasing use of RHIS for research and evaluative purposes, though underused for HIV/AIDS.^5^,^8^ RHIS typically has aggregate counts of health service output data at facility levels, while surveys produce very robust but narrow epidemiological parameters for specific research objectives. RHIS data are routinely available in almost every high-burden HIV setting, and there exists a great opportunity to leverage these routinely collected data for cost-effective secondary analyses to inform HIV policy and programmatic decision-making.^9^

In Malawi, a major RHIS is the District Health Information Software-2 (DHIS2), a system that tracks quarterly facility-level data across specific disease areas such as HIV, sexually transmitted infections (STIs), tuberculosis and malaria, as well as for general health programmes such as disease surveillance, routine immunisation and maternal and child health.^10^ Given that HIV and other STIs have similar modes of transmission, although with differing presentations (and probabilities), a rapid increase in syndromic STIs in a specific geographical area could be signals of sexual risk and therefore could be bellwethers for undiagnosed HIV.^11^ Especially as individuals with STIs are at an increased risk of transmitting and acquiring HIV,^12^ many studies focus on the need for programmatic change to integrate STI services with HIV in a population. Few studies, however, examine STI diagnosis trends with other contextual factors (ie, geography, facility type, facility ownership) in understanding and describing HIV positivity across the population. We sought to leverage routinely collected programmatic data at the facility level to describe and predict changes in HIV test positivity at the subdistrict level in Malawi.

## Methods

### Data sources

This project used quarterly data from Malawi’s DHIS2 from 2017 Q1 (January–March 2017) to 2023 Q1 (January–March 2023) as well as its 2016 Population-Based HIV Impact Assessment (PHIA) data. DHIS2 variables extracted included HIV tests and test results, syndromic STI counts (syphilis, urinal discharge, lower abdominal pain, scrotal swelling, genital ulcers, high-risk, vaginal discharge) and contextual covariates such as health facility type (hospital, health centre, dispensary) and ownership (faith-based, private, public), quarter, season (rainy, dry). PHIA variables include zonal prevalence (the first subnational administrative level) of HIV, viral load suppression, genital discharge, genital ulcers and clinical STI diagnoses.

From the 713 health facilities across all 28 districts nationwide in Malawi reporting STI and HIV Testing Clinic data, we included facilities reporting 75% or more of reporting quarters. This led to an analytic sample of 563 facilities that represented 86% of HIV tests administered. Among this sample, missing data entries were imputed with facility averages.

### Analysis

We hypothesised that the association between changes in syndromic STI counts and changes in HIV testing positivity is dependent on the type of STI and their incubation period; STI care-seeking behaviour; HIV testing guidelines and test utilisation; lagging analysis for specific STIs; high-quality facility-level data and sufficient temporal variability in both STI and HIV diagnoses.

Depending on the type of syndromic STI and its respective incubation and symptomatic period, an HIV test done at the same time may or may not detect a new HIV infection using a fourth-generation HIV rapid test. Thus, the type of syndromic STI and their incubation and syndromic periods were considered in the analysis (online supplemental appendix A). Syndromic STIs that may have a simultaneous signal of HIV with a rapid test were compared with HIV positivity in the same quarter (syphilis, abdominal pain and urethral discharge). Conversely, syndromic STIs such as scrotal swelling, genital ulcers and vaginal discharge were compared with HIV diagnoses four quarters later, as these STIs have a shorter incubation and symptomatic period. These syndromic STIs would not have a positive HIV rapid test during the STI treatment, even if the individual had recently contracted HIV—they would, however, show a positive test on a subsequent referral for testing. While the full descriptive dataset encompassed data from 2017 Q1 to 2023 Q1, the analytic dataset with the lagged STI counts started from 2018 Q1.

We used a multilevel, beta-regression model to assess our primary outcome of HIV test positivity, a ratio of the number of positive tests to the total tests administered. We accounted for clustering and auto-correlation providing random slopes for facility and geographic zone. In our multilevel model, our primary exposure variables were the lagged and cross-sectional syndromic STI counts. We further controlled for contextual variables such as health facility type (hospital, health centre, dispensary) and ownership (faith-based, private, public), quarter, season (rainy/dry), zonal HIV and clinical STI prevalence, and weighted by the total number of HIV tests. We ran this model across three, seven-quarter time periods between 2018 Q1–2023 Q2 (period 1: 2018 Q1–2019 Q3; period 2: 2019 Q4 –2021 Q2; period 3: 2021 Q3–2023 Q1). We recognise 2020 Q2 (April–June 2020) in our data as just after the start of the COVID-19 pandemic and approximately when disruptions in the healthcare system would have occurred. Therefore, by creating these three time periods, we can isolate trends related to the COVID-19 pandemic within period 2.

All analyses were conducted using R V.4.2.1 (www.r-project.org)

### Patient and public involvement

It was not appropriate or possible to involve patients or the public in our research’s design, conduct, reporting, or dissemination plans.

## Results

### Facility characteristics

Among the 563 facilities included in this analysis, there were 70 (12%) dispensaries, 397 (71%) health centres and 73 (13%) hospitals (table 1). Publicly owned facilities (388; 69%) were the majority, followed by faith-based organisations (138; 25%) and privately owned facilities (37; 6.6%). Blantyre zone had the least number of health facilities included in this analysis (n=33), while the south-east zone had the greatest number of facilities (n=104). Most facilities were in rural locations (467; 83%).

**Table 1:** HIV test counts, STI counts and HIV positivity by facility characteristics during the study period, 2018 Q1–2023 Q1 (full table including time period breakdowns is provided in online supplemental appendix B)

	Total HIV tests	HIV positivity	Cross-sectional STI counts	Lagged STI counts
Total (n=563)	13 947 245	0.028	847 739	555 567
Facility type				
Dispensary (n=70)	1 309 885	0.034	94 058	57 760
Health centre (n=397)	8 194 799	0.027	454 473	302 255
Hospital (n=73)	3 969 531	0.027	268 949	176 285
Other (n=23)	473 030	0.035	30 259	19 267
Ownership				
Faith-based (n=138)	2 740 064	0.028	122 346	73 697
Private (n=37)	581 699	0.030	35 743	21 427
Public (n=388)	10 625 482	0.028	689 650	460 443
Residence				
Missing (n=30)	614 221	0.023	32 700	23 340
Rural (n=467)	9 737 174	0.026	540 457	349 359
Urban (n=66)	3 595 851	0.034	274 582	182 868
Season				
Dry	6 588 578	0.028	399 045	275 315
Rainy	7 358 667	0.028	448 694	280 252
Zone				
Blantyre (n=33)	745 973	0.055	61 550	41 271
Central-east (n=91)	2 067 741	0.017	113 527	75 289
Central-west (n=74)	1 574 175	0.023	98 470	70 293
North (n=99)	1 334 424	0.024	87 761	57 493
South-east (n=114)	3 224 813	0.029	158 752	97 070
South-west (n=104)	2 870 120	0.032	208 700	135 983

STI, sexually transmitted infection.

### Current HIV and STI trends in Malawi

**Figure 1:**
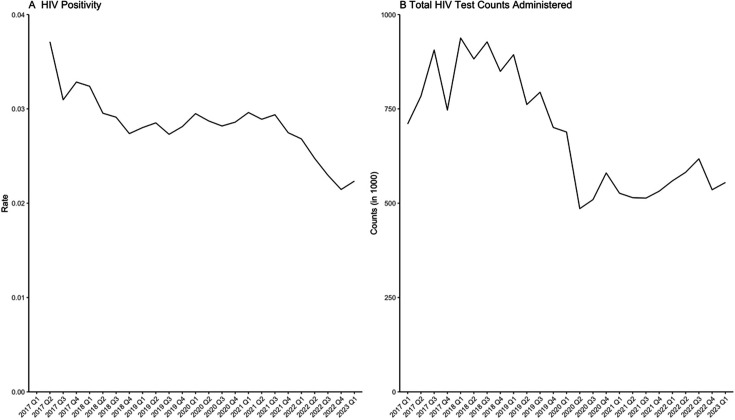
Quarterly (A) HIV positivity and (B) positive and negative HIV test counts (thousands), April 2017–March 2023.

**Figure 2:**
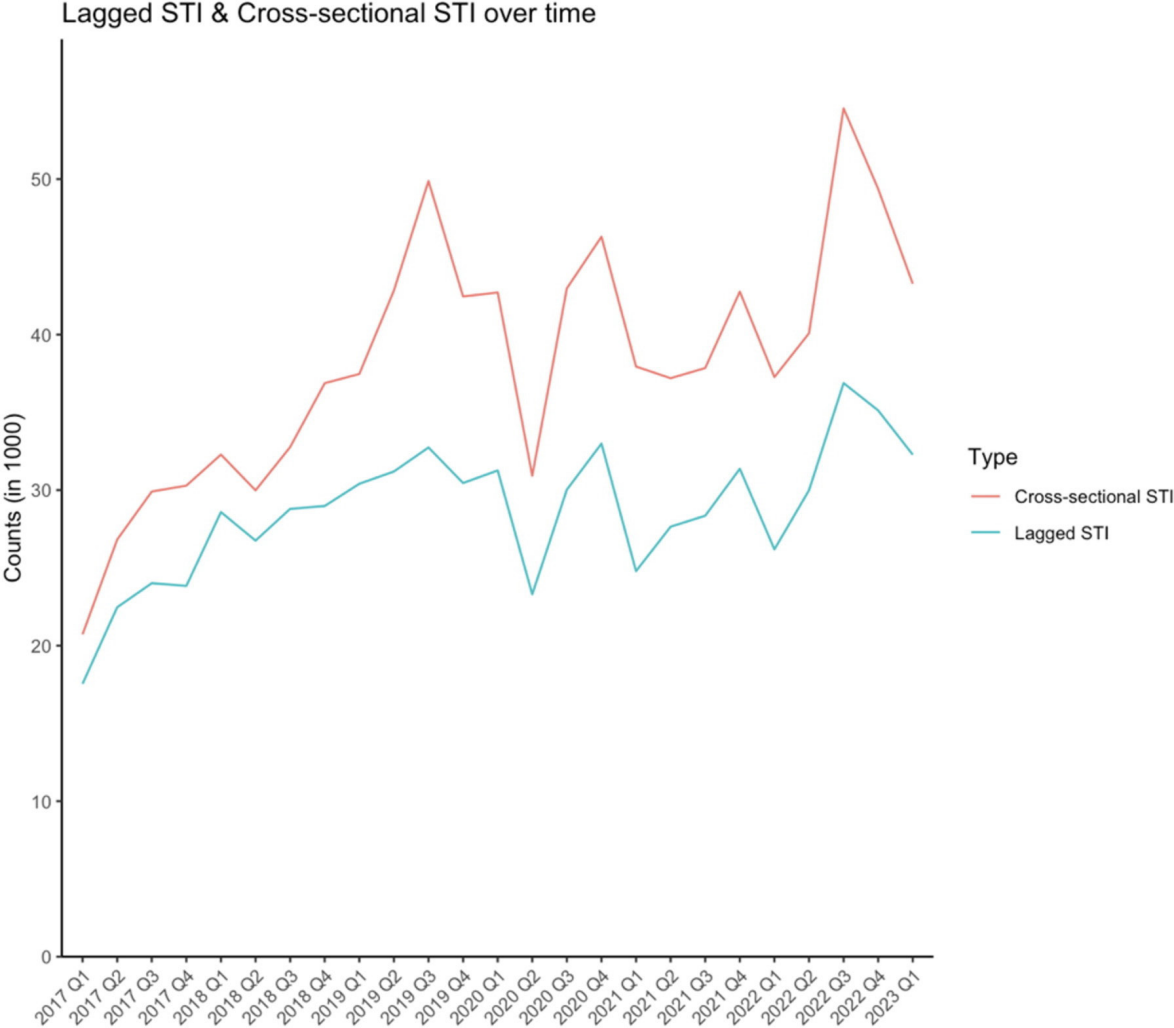
Lagged sexually transmitted infection (STI) and cross-sectional STI counts (thousands), April 2017–March 2023.

Cumulatively across the study period, the overall HIV test positivity was 2.8% (table 1) and declined over time. We observed a plateau in positivity from 2018 to 2021 before it declined through 2022 (figure 1A). The total HIV test counts have also decreased, with a sharp decline in 2020 Q2, with little rebound (figure 1B). On the other hand, total counts of STI, both for lagged and for cross-sectional STIs, increased slightly throughout the study period (figure 2). There is a visible drop in STI counts in 2020 Q2 but the pattern recovered and continued in the quarter after.

**Figure 3:**
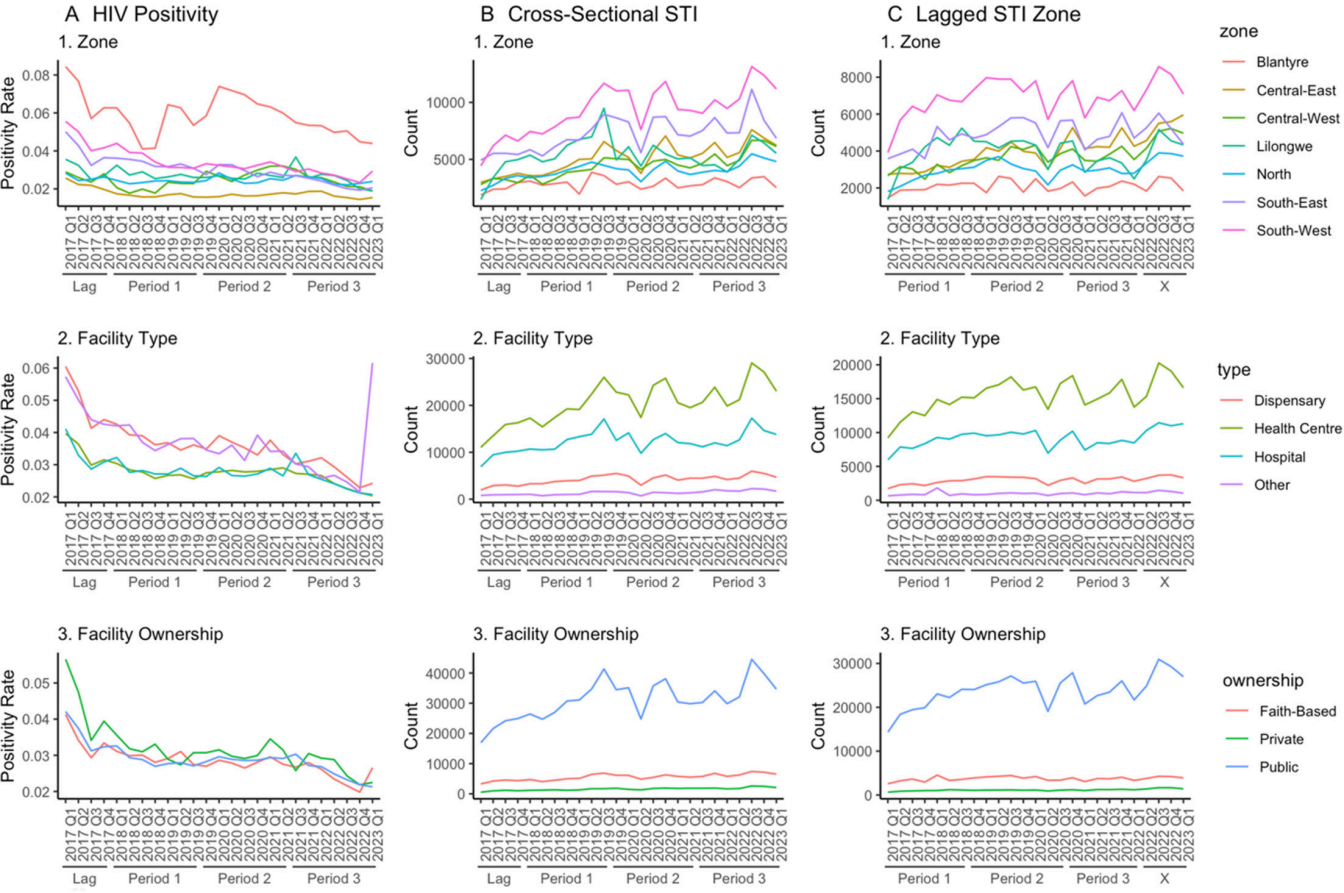
Panel showing (A) HIV positivity, (B) lagged sexually transmitted infection (STI) counts and (C) cross-sectional STI counts disaggregated by contextual factors. 1, zone; 2, facility ownership; 3, facility type; X, because of the lag in STI counts, these four quarters are not included in the regression analyses.

Disaggregated by facility factors (zone, facility type and facility ownership), we observed somewhat consistent patterns over time, where HIV positivity decreased and STI diagnoses increased with a temporary dip in 2020 Q2 (figure 3). Blantyre zone had the fewest number of HIV tests administered across all three time periods but had the highest HIV positivity (table 1, online supplemental appendix B, figure 3). Blantyre’s HIV positivity pattern is different from the other zones, with a dramatic spike in HIV positivity in 2020 Q1 before a steady decrease. This pattern is reflected across the three analytic periods, with Blantyre’s HIV positivity at 5.2% in period 1, increased to 6.5% in period 2 and then decreased back to 5.0% in period 3. Total HIV tests have decreased across all three time periods as well, with the first period having 6 million tests administered, but by the last period, that number decreased to 3.9 million (online supplemental appendix B). Across the facility ownership category, there was not much difference in HIV positivity; however, when examining facility type, HIV positivity was highest in dispensaries (table 1, figure 3). HIV positivity for facility type ‘Other’ seems to be anomalous in 2023 Q1, which may be due to there being only 23 facilities out of 563 in this category.

### Multivariable regression results

We first conducted a multilevel beta-regression model for the entire analytic period of 2018 Q1–2023 Q3, with detailed results in table 2. We saw that our main parameters of interest, quarterly changes in lagged and cross-sectional syndromic STI counts, do not have strong associations with HIV positivity, with smaller effect sizes relative to other associations in our model (OR: 1.01, CI: 1.01 to 1.01; OR: 1.00, CI: 1.00 to 1.00). However, contextual covariates such as the zonal prevalence of HIV (OR: 1.04, CI: 1.04 to 1.04), genital ulcers (OR: 1.16, CI: 1.16 to 1.16) and clinical STI diagnoses (OR: 1.29, CI: 1.29 to 1.29) were positively associated with HIV positivity across the three time periods. Conversely, increasing zonal prevalence of viral-load suppression (OR: 0.96, CI: 0.96 to 0.96) and zonal prevalence of genital discharge (OR: 0.66, CI: 0.66 to 0.66) were associated with lower HIV positivity. Among those contextual covariates, zonal prevalence of clinical STI diagnoses had the greatest association with HIV positivity, with a 1% increase in STI diagnoses associated with a 28.8% increase in HIV positivity controlling for all other covariates.

**Table 2:** Results of the multivariable beta-regression examining associations with HIV positivity, exponentiated

Parameter	Period 1 OR (95% CI)	Period 2 OR (95% CI)	Period 3 OR (95% CI)	Entire time period OR (95% CI)
Quarterly change in lagged syndromic STIs (per 100)	0.980 (0.980 to 0.980)	0.997 (0.997 to 0.998)	1.010 (1.009 to 1.011)	1.0078 (1.007 to 1.008)
Quarterly change in cross-sectional syndromic STIs (per 100)	1.021 (1.021 to 1.021)	0.992 (0.992 to 0.992)	0.997 (0.997 to 0.997)	0.999 (0.999 to 0.999)
Zonal contextual covariates				
Prevalence of HIV (per 1%)	1.057 (1.057 to 1.057)	1.036 (1.036 to 1.036)	1.028 (1.028 to 1.028)	1.043 (1.042 to 1.043)
Prevalence of viral-load suppression (per 1%)	0.980 (0.980 to 0.980)	0.948 (0.948 to 0.948)	0.951 (0.951 to 0.952)	0.962 (0.962 to 0.962)
Prevalence of genital discharge (per 1%)	0.702 (0.700 to 0.704)	0.547 (0.546 to 0.549)	0.677 (0.675 to 0.679)	0.661 (0.990 to 0.662)
Prevalence of genital ulcer (per 1%)	1.136 (1.135 to 1.137)	1.200 (1.204 to 1.206)	1.182 (1.181 to 1.183)	1.164 (1.163 to 1.164)
Prevalence of clinical STI diagnosis (per 1%)	1.231 (1.230 to 1.232)	1.459 (1.459 to 1.461)	1.27 (1.268 to 1.272)	1.288 (1.286 to 1.289)
Quarter	0.987 (0.987 to 0.988)	0.999 (0.999 to 1.000)	0.959 (0.959 to 0.960)	0.99 (0.990 to 0.990)
Season				
Dry	Ref.	Ref.	Ref.	Ref.
Rainy	0.964 (0.963 to 0.965)	1.004 (1.003 to 1.005)	1.021 (1.020 to 1.022)	0.994 (0.993 to 0.994)
Facility type				
Dispensary	Ref.	Ref.	Ref.	Ref.
Health centre	0.819 (0.817 to 0.820)	0.825 (0.823 to 0.826)	0.900 (0.900 to 0.902)	0.849 (0.848 to 0.850)
Hospital	0.990 (0.988 to 0.992)	0.941 (0.939 to 0.943)	1.015 (1.012 to 1.017)	0.990 (0.989 to 0.991)
Other	1.017 (1.015 to 1.020)	1.097 (1.094 to 1.101)	1.135 (1.131 to 1.138)	1.078 (1.076 to 1.080)
Facility ownership				
Faith-based	Ref.	Ref.	Ref.	Ref.
Private	0.879 (0.969 to 0.873)	1.038 (1.034 to 1.041)	1.055 (1.052 to 1.058)	0.978 (0.976 to 0.980)
Public	0.985 (0.984 to 0.986)	1.038 (1.037 to 1.039)	1.046 (1.044 to 1.047)	1.018 (1.018 to 1.019)

Ref., reference; STI, sexually transmitted infection.

For each progressing quarter, on average, HIV positivity falls just by 1% when controlling for all covariates. HIV positivity is also lower in the rainy season—perhaps due to care-seeking—by 0.6% (OR: 0.994; CI: 0.993 to 0.994). Examining the health facility types, health centres have a lower HIV positivity relative to dispensaries by 15%; whereas hospitals and other facility types have similar positivity to dispensaries. When considering health facility ownership, private facilities have lower positivity relative to faith-based organisations by 2.2%, whereas public facilities have higher positivity relative to faith-based facilities by 1.8%.

Across the three, seven-quarter periods, we see that the zonal contextual covariates and their associations with HIV positivity stay consistent from the models. Zonal prevalence of HIV, genital ulcer and clinical STI diagnoses are positively associated, whereas the prevalence of genital discharge and viral-load suppression is inversely associated with HIV positivity. While we saw that quarterly changes in syndromic STI (lagged and cross-sectional) were not strongly associated with HIV positivity when considering the full analytic period, when examining across the different time periods, we observed heterogeneity in the associations. With the lagged STIs, we see that a quarterly increase of 100 STIs is associated with a 2% decrease in HIV positivity in period 1, but a 1% increase in HIV positivity in period 3. For cross-sectional STIs, period 1 saw a 2% increase in HIV positivity for a quarterly change of 100 diagnoses, but no association in period 2 or 3. While the associations between facility type and HIV positivity mostly stay consistent across the time periods, the associations between facility ownership and HIV positivity do not.

## Discussion

We analysed Malawi’s HIV positivity trend over the past 6 years using available RHIS data and explored the contextual factors associated with the changes in positivity at the facility level. While we found heterogeneous results from our primary syndromic STI parameters of interest, we nonetheless observed interesting patterns across contextual factors such as facility type, facility ownership, rainy or dry season and zonal HIV and clinical STI prevalence. For facilities in zones that differ in prevalence of clinical STI diagnosis by 1%, there is a 23% difference in programmatic HIV test positivity between those facilities, all else equal. Similarly, a 1% difference in zonal prevalence of genital ulcers was associated with a 16.4% increase in HIV positivity, controlling for all considered covariates.

The results are consistent with prior research that identified an increase in HIV acquisition among those already with STIs, as well as with studies that identified opportunities to improve the concurrent testing of HIV at STI clinics.^11 13 14^ Understanding these contextual trends and associations over time through programmatic data can inform HIV prevention-related resource allocation and prioritisation processes in the future. Additionally, our results, especially ones showing how HIV test counts decreased dramatically after the first quarter of 2020, are consistent with current research on the health system effects of COVID-19. An interrupted time-series analysis in Malawi showed that HIV tests declined by 31.9%, and the number of PLHIV diagnosed declined by 22.8%, immediately after non-pharmaceutical COVID-19 restrictions were put in place.^15^ Globally, studies have shown similar results regarding disruptions in HIV testing services as well as pre-exposure prophylaxis (PrEP) uptake and treatment adherence. Systematic reviews have found a 37% decrease in the HIV testing rate during the pandemic years compared with before 2019, and that individuals in PrEP care stopped using PrEP or indicated challenges in receiving care.^16 17^ In-person medical visits and adherence to HIV treatment had been interrupted as well, with subsequent increases in psychological disorders, substance abuse, and stigma and discrimination impacting care-seeking behaviours.^18^ Augmented efforts are needed to return to pre-pandemic levels of health system capacity in order to effectively be able to identify and treat those living with HIV.

The results of this study have important implications for monitoring and priority setting of HIV programme services across the health system, and may be especially beneficial in supporting Malawi’s existing national HIV testing strategies, including index and community-based testing, by identifying geographic or facility-level hotspots for prioritisation. We found that trends in HIV positivity are transient, difficult to predict and require contextual data inputs. Thus, large-scale programmes, rather than simply relying on past HIV positivity for future prioritisation and decision-making purposes, may consider understanding contextual factors, including STI diagnosis trends. For example, in our analysis, we found greater zonal prevalence of clinical STI diagnoses was associated with increases in HIV positivity, and therefore facilities and regions where clinical STI diagnoses are high may receive prioritisation in terms of HIV testing outreach. Though many of the ORs in our model were statistically significant, some reflected small effect sizes (eg, OR: 1.01). These small increases may have limited clinical meaning when interpreted in isolation. However, in the context of relative trends and when used alongside stronger contextual predictors such as zonal HIV prevalence or clinical STI diagnoses, even small shifts in syndromic STI trends may still hold programmatic value for tracking patterns or guiding targeted interventions. Using programmatic, routinely collected data to inform trends in HIV positivity may be of importance—programmes in place already trying to reduce the burden of HIV using data may integrate this to enhance their outreach, testing, prevention and treatment services as part of their priority-setting processes.

An already well-used system provides a great opportunity for the integration of a model assessing contextual covariates to determine which facilities may experience an uptick in HIV-positive tests. The Blantyre Prevention Strategy in Malawi is a systems-based approach to HIV prevention, aimed at using data to target risk and deliver HIV prevention services and interventions.^19 20^ One of the key components of this programme is a user-centred dashboard from a data pipeline that displays real-time facility-level trends across participating facilities and helps enable linking STIs and HIV diagnoses for responders. As this system displays multiple routinely collected programmatic data, incorporating an identifiable flag or notification system that is based on statistical models could help healthcare workers plan and prepare for an uptick in patients.

Finally, these results should be interpreted in the context of the study’s limitations. First, syndromic STI management does not capture all incident STIs, which may attenuate effect size. Care-seeking behaviour and test utilisation may vary, and some individuals may not return for HIV testing after an STI diagnosis, reducing the ability to detect temporally linked HIV cases. The lagged effect in the model also oversimplifies care-seeking and guidance adherence and assumes a uniform timeline, which may reduce the effect size. Our data also include recurrent STIs, while HIV positivity reflects new HIV-positive test results. Additionally, the predictive value of syndromic STI trends may be limited, particularly in the absence of laboratory-confirmed diagnoses. Syndromic diagnosis relies on clinical judgement and may be open to misclassification bias, highlighting the potential for integrating laboratory-confirmed diagnoses and testing systems for future studies.

Second, reporting bias and data quality issues at the facility level may affect our findings. While our analysis excludes facility data that have lower than a threshold value of data reporting and imputes the remaining missing data, using routine data is inevitably susceptible to underreporting or poor-quality data. Differences in reporting practices between facility types or zones may also contribute to systematic bias. In addition, using programmatic data in this way does open threats to validity, specifically from co-occurring events such as HIV prevention and treatment programmes throughout the country that have been adopted throughout the study period. Importantly, we cannot determine the causal factors affecting HIV test positivity, some of which may include targeted testing approaches or challenges in testing programmes. We also acknowledge the limitations of aggregate, health facility-level data, as individual-level data and further sensitivity analyses could indeed closely examine the temporal relationships between STI diagnoses and HIV testing outcomes. Nevertheless, this study provides an additional method for understanding trends in HIV positivity across a population, specifically using readily available, large-scale, aggregate routine health data.

## Conclusions

In settings with high uptake of STI screening, assessing trends in STI diagnoses, along with considering contextual factors such as facility type, facility ownership, seasonality and zonal HIV and clinical STI prevalence, could inform the identification of HIV hotspots so that they can be accompanied by appropriate testing, prevention and treatment services. Furthermore, using already existing national, routinely collected health data is important as it allows for efficient analyses and evaluation of programme areas of interest within the decision-making processes at all levels of the health system. Our study provides the foundation for subsequent studies to build upon, perhaps towards the use of machine learning techniques to predict hotspots and integrate a real-time system to flag the geographic districts or facilities that would potentially experience an increase in HIV positivity. Given that HIV positivity is low and decreasing, any predictive capability may have important programmatic implications.

## Supplementary material

10.1136/bmjph-2025-002568 online supplemental appendix 1

10.1136/bmjph-2025-002568 online supplemental appendix 2

## Data Availability

Data may be obtained from a third party and are not publicly available.
