# Supplementary material for: Health facility and contextual correlates of HIV test positivity: a multilevel model of routine programmatic data from Malawi
Source: BMJ Public Health. 2025 Sep 8;3(2):e002568. doi: 10.1136/bmjph-2025-002568 (PMC12421179; doi:10.1136/bmjph-2025-002568)

**Appendix A.** Conceptual Framework for STI Lag Period and HIV Test Result: (a) Lagged STI and (b) Cross-sectional STI


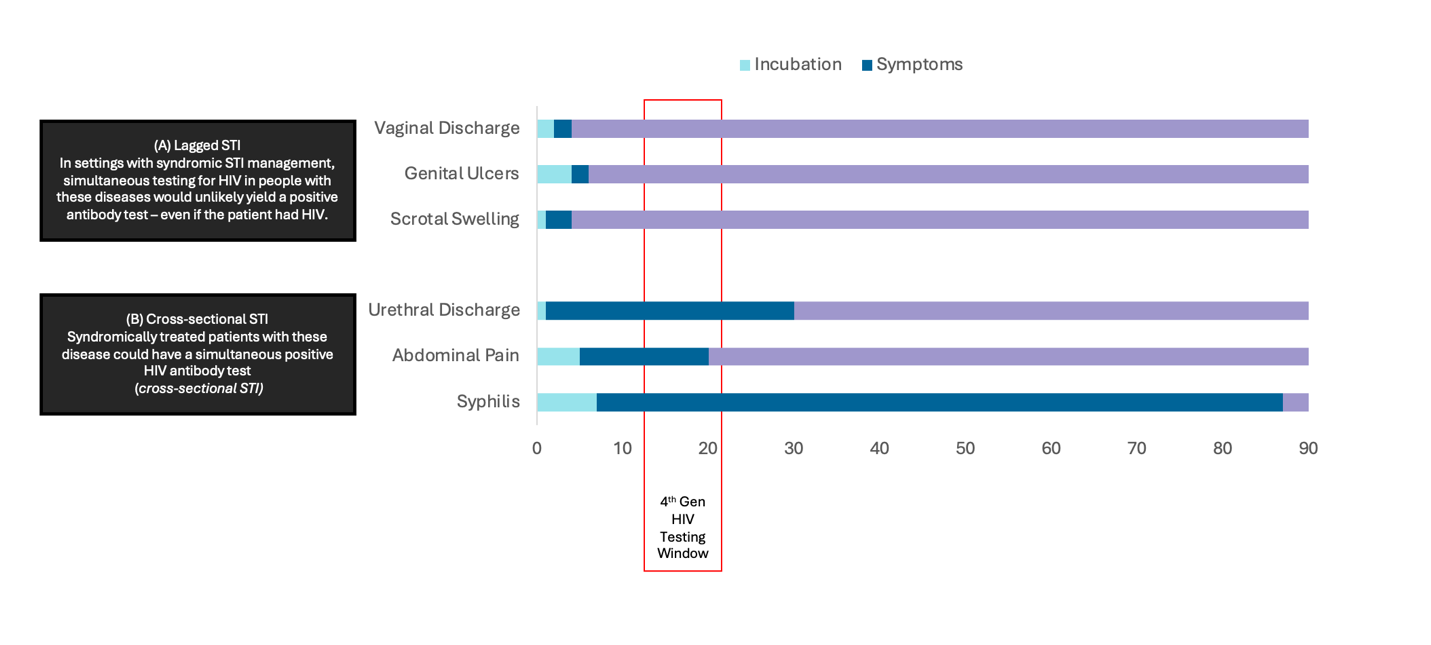

Supplement: online supplemental appendix 1 [file bmjph-3-2-s001.docx]
