# Supplementary material for: Health facility and contextual correlates of HIV test positivity: a multilevel model of routine programmatic data from Malawi
Source: BMJ Public Health. 2025 Sep 8;3(2):e002568. doi: 10.1136/bmjph-2025-002568 (PMC12421179; doi:10.1136/bmjph-2025-002568)
Supplement: online supplemental appendix 2 [file bmjph-3-2-s002.pdf]

Appendix B. Extension of Table 1: HIV test counts, STI counts, and HIV Positivity by Facility Characteristics and Time Period. Period 1 (P1): 2018 Q1–2019 Q3; Period 2 (P2): 2019 Q4 –2021 Q2; Period 3 (P3): 2021 Q3 –2023 Q1); Full: Full Time Period (2018 Q1 – 2023 Q1).

	**Total HIV Tests**				**HIV Positivity**				**Cross-Sectional STI Counts**				**Lagged STI Counts**			
	**P1**	**P2**	**P3**	**Full**	**P1**	**P2**	**P3**	**Full**	**P1**	**P2**	**P3**	**Full**	**P1**	**P2**	**P3**	**Full**
**Total (N= 563)**	**6046432**	**4005980**	**3894834**	**13947245**	**0.029**	**0.029**	**0.025**	**0.028**	**262094**	**280499**	**305146**	**847739**	**172018**	**238389**	**238389**	**555567**
**Facility Type**																
Dispensary (n=70)	544548	382028	383309	1309885	0.038	0.036	0.028	0.034	28271	31619	34168	94058	17021	25228	25228	57760
Health Centre(n=397)	3523258	2345572	2325969	8194799	0.027	0.028	0.024	0.027	136966	152663	164844	454473	90536	130608	130608	302255
Hospital (n=73)	1786285	1144977	1038270	3969531	0.028	0.027	0.025	0.027	88890	87100	92959	268949	57888	75053	75053	176285
Other (n=23)	192341	133403	147286	473030	0.038	0.035	0.031	0.035	7967	9117	13175	30259	6573	7500	7500	19267
**Ownership**																
Faith-Based (n=138)	1158538	812490	769036	2740064	0.030	0.028	0.025	0.028	36594	40139	45613	122346	23760	31479	31479	73697
Private (n=37)	236278	174885	170536	581699	0.031	0.031	0.026	0.030	9532	11916	14295	35743	6782	8699	8699	21427
Public (n=388)	4651615	3018605	2955262	10625482	0.029	0.029	0.025	0.028	215968	228444	245238	689650	141476	198211	198211	460443
**Residence**																
Missing (n = 30)	259163	179268	175789	614221	0.025	0.025	0.020	0.023	10241	10700	11759	32700	7724	9701	9701	23340
Rural (n=467)	4187391	2790403	2759380	9737174	0.027	0.027	0.024	0.026	156565	180213	203679	540457	104976	150795	150795	349359
Urban (n=66)	1599877	1036308	959665	3595851	0.035	0.035	0.030	0.034	95288	89586	89708	274582	59318	77893	77893	182868
**Season**																
Dry	3365608	1509815	1713155	6588578	0.029	0.029	0.026	0.028	155463	111099	132483	399045	102033	117279	117279	275315
Rainy	2680824	2496165	2181678	7358667	0.029	0.029	0.025	0.028	106631	169400	172663	448694	69985	121110	121110	280252
**Zone**																
Blantyre (n =33)	335039	197248	213686	745973	0.052	0.065	0.051	0.055	21077	19393	21080	61550	13739	17202	17202	41271
Central-East (n=91)	882871	608480	576390	2067741	0.017	0.017	0.017	0.017	32064	37974	43489	113527	20961	31222	31222	75289
Central-West(n=74)	745679	420798	407698	1574175	0.021	0.029	0.024	0.023	26791	32754	38925	98470	20639	30519	30519	70293
North (n=99)	581911	394170	358343	1334424	0.024	0.025	0.024	0.024	28237	27879	31645	87761	17810	24698	24698	57493
South-East (n=114)	1322544	916264	986006	3224813	0.033	0.029	0.023	0.029	47305	54167	57280	158752	29980	41854	41854	97070
South-West (n=104)	1200007	849444	820669	2870120	0.036	0.032	0.028	0.032	61957	70997	75746	208700	42579	58877	58877	135983
